# Supplementary material for: Feasibility and acceptability of using the BabySaver resuscitation platform and NeoBeat together for neonatal resuscitation in a low-resource setting: A pre-post implementation study
Source: PLoS One. 2025 Dec 4;20(12):e0337088. doi: 10.1371/journal.pone.0337088 (PMC12677507; doi:10.1371/journal.pone.0337088)
Supplement: S1 Table — TFA = Theoretical Framework on Acceptability. (DOCX) [file pone.0337088.s001.docx]

| TFA construct | Finding (subtheme) | Meaning unit |
| --- | --- | --- |
| Affective attitude:  This implies how an individual feels about the intervention | BabySaver and NeoBeat use simplifies neonatal resuscitation  Resuscitation at the bedside with an intact cord is time saving  BabySaver provides a clean environment for resuscitation  NeoBeat use during resuscitation motivates the health worker  Builds trust between health workers, patients and caregivers  Patient satisfaction  Reassurance to the mother  Coping with loss is smoother  Resuscitating at the bedside motivates the health worker to do better | *“This new era of NeoBeat and resuscitation platform, it can be done without an assistant. It's even easier and not cumbersome” HW 008*  *“When I'm resuscitating, the Neobeat will actually be directing me if I'm doing it right or I'm doing it wrong, or if I have to adjust the what? Adjust the mask or the baby's neck. Yeah, so to my experience, I see the Neobeat and the baby saver are making my work like simpler than before…..* *Because I don't have to start again touching the stethoscope to listen to the heartbeat. So I'll be focusing on my resuscitation as I'm looking at the readings from the Neobeat.” HW 004*  *“I felt it was so easy. Because, first of all, it saves time. And I don't waste time to tie the cord and transfer the baby to the other side.” HW 007*  *“You know the BabySaver provides a clean environment for resuscitation. Because you cover with a sheet, you put the baby, and then you cover on top and just expose the chest. You have covered the baby's head.” HW 001*  *“You can't lose hope. You see the heart rate showing, improving. So, you say, the baby is improving. I should not give up. So, it's really very good.” HW 001*  *“It will help you to know whether your resuscitation is a success or you should abandon it. If you are resuscitating and you see the fetal heart is improving, decreasing, it motivates you to say, I think this baby is still alive.” HW 006*  *“For me as a health worker, I advocate for it because many of us have gone.… to court because of false accusations. They exchanged my baby. They have killed my baby. Maybe Musawo (health worker) was not minding. So, when they are there, I think that it's also better the health worker. For me, if it is me, whether you consent or not, I have to resuscitate from there because I need to safeguard myself.” HW 010*  *“I don't think it's a challenge because the mother gets to know what is going on. Like in, even if the baby dies, like she will not say she was neglected, Musawo (health worker) tried.” HW 004*  *“There is that kind of the relationship between the health worker and the mother because why do I say so? The mother really will see you struggling and the baby came up. Of course, the mother will have to say thank you.” HW 003*  *“I feel good because while the mother is observing the baby, she is reassured that the baby is going to be fine than taking away the baby to some other place.” HW 007*  *“Yes. Because it gives you a sense of... Saying my baby is coming slowly, slowly. Even if things don't turn out good, you'd be okay that that was my baby. Yeah. They have not changed; they have not done anything wrong. It was God's plan. Okay.” Mother 002*  *“Even if the baby dies, actually breaking out the news, it becomes so easier for you compared when you resuscitate this baby in a very far place or in a different way. And even in a bereavement of a mother, she will start building up from when she's observing how the baby is trying to cope up with life. And I think in bereavement it becomes easier for the mother to cope up and say, they tried to do this ABCD and the baby failed.” HW 010*  *“you always feel guilty to do your best because the mother is observing.” HW 003* |
| Burden and self-efficacy  Burden: The perceived amount of effort required to participate in the intervention  Self-efficacy: Participant’s confidence that they can perform behavior required by intervention | The BabySaver and the NeoBeat are easy to use | *“It doesn't need a lot of skills, it is flat, and it has that position for the head where we can put the baby in a sniffing position to make the airway pattern such that you are able to bag and you are able, the baby is able to take in what? The breath that you are delivering.” HW 009*  *“Putting the NeoBeat is very easy because we able to get the baby's heart rate very fast without looking for the pericordium or the cord for us to start looking out to count the heart rate.” HW 008*  *“It is a portable device, which is very good. And it is easy to attach and detach if needed. And also, it has, it is wireless, you know? Like that is easier, you don't need to connect it to power when you are resuscitating.” HW 008* |
| Intervention Coherence and perceived effectiveness  Intervention coherence: Extent to which the participant understands the intervention and how it works  Perceived effectiveness: Extent to which intervention is perceived to achieve its purpose | BabySaver provides a flat surface for resuscitation  Bedside resuscitation is effective: Improved birth outcomes | *“it gives us that flat surface whereby you can place (the baby) and it has a design whereby even you can put the head of the baby for positioning. It mainly provides that surface for easy maneuvering.” HW 003*  *“It is really effective. The babies cry after some time, after resuscitation. And even, most of them we don't send them to the neonatal unit. They pick up very early.” HW 001* |
| Opportunity costs and Ethicality  Opportunity costs: Extent to which benefits, profits, or values must be given up to engage in the intervention  Ethicality: Extent to which the intervention has good fit with an individual’s value system | Cultural beliefs, myths and misconceptions: Mothers blood attacks the baby, Fear of HIV transmission:  Fear of hypothermia: Covering the baby isn’t enough  Perceived risk for cross infections  Difficult to use the BabySaver in theatre | *“Yeah. You're supposed to cut immediately. Even though that the last one (placenta) has not come, they need to cut the cord faster. Quickly, quickly. That they don't want blood of the mother to attack the baby.” Mother 002*  *“Some people say that the baby dies in case you don’t cut the cord immediately. There in the villages even in the health facilities they say that once the baby is born and cries you have to cut the cord immediately otherwise the baby might die.” Mother 012*  *“But it will depend on which patient you are dealing with. Because for a mother with HIV, I cannot do it, I cannot wait for all that time. This baby should be separated immediately from the mother, and to avoid the vertical transmission of HIV/AIDS.” HW 009*  *“The worst part of it is I think the warmth, but the rest is okay. Even if we cover. They come up from hibernation so they need a warmer. We try to cover but still. ..That's the only thing maybe.” HW 002*  *“Yes, alcohol is very good because it dries off very fast. But however, I don't know whether it kills all the microbes on it. Like I said, some other babies come up with different Meconium. Meconium, yes, chorioamnionitis, so that's where my mind can hesitate” HW 003*  *“Barriers is there is no space. The space is limited. Now, it might even contaminate the area of the surgery.” HW 003*  *“As I told you, in theatre, we still have a challenge. Implementing it the way we use it in the labor ward. Because the mother is in a recumbent position. They tie somehow the legs. They want them to be together.” HW 001*  *“And here, even if you have performed a spinal anaesthesia, they are not able to see. But you only explain to them, you know, the baby has come out but is not doing well and we are trying our best to make sure the baby improves on this BabySaver. I would like the mother to see what is going on. But due to the nature of the procedure we need the environment to be sterile.” HW 009* |
