# Supplementary material for: Feasibility and acceptability of using the BabySaver resuscitation platform and NeoBeat together for neonatal resuscitation in a low-resource setting: A pre-post implementation study
Source: PLoS One. 2025 Dec 4;20(12):e0337088. doi: 10.1371/journal.pone.0337088 (PMC12677507; doi:10.1371/journal.pone.0337088)
Supplement: Attachment — Submitted filename: DCC_response to reviewers.docx [file pone.0337088.s003.docx]

**Re: “PONE-D-25-16948 Feasibility and acceptability of using the BabySaver resuscitation platform and NeoBeat together for neonatal resuscitation in a low-resource setting: A pre-post implementation study”**

Thank you for revising our manuscript.

Below is our point-by-point response to each comment.

Thank you.

| **Comment** | **Response to comment** | **Line number** |
| --- | --- | --- |
| **Editor** |  |  |
| 1. Please correct the author affiliations: The author affiliations do not match - “9” and “11” are not assigned to an author. | Thank you for pointing this out. We have reviewed and corrected the author affiliations. The numbers “9” and “11” have now been appropriately assigned to the relevant authors, and all affiliations are now correctly matched. | 3-6 |
| 2. Please add further details to the funding, conflict of interest sections, and the text in the manuscript, to make appropriate reference to the website which describes the work of Andrew Weeks (the last author): https://safri.org/the-baby-saver/   The website states:  The BabySaver, developed by Professor Andrew Weeks, a consultant obstetrician from Liverpool.  The product has been developed in conjunction with Peter Watt, a design engineer at the Royal Liverpool and Broadgreen University Hospitals Trust, after trials with staff and patients at the Mbale Regional Referral Hospital (MRRH) in Uganda.  The prototypes have been manufactured by a team at the Bryn Y Neuadd Hospital in Gwynedd.  The project has been funded by Grand Challenges Canada, and the Sir Halley Stewart Trust.  NOTE:  i) Since Andrew Weeks is the designer/developer of the device which is being tested, that should be stated, as well as a clear statement regarding the agreements between him and the funders, including subsequent renumeration for sales of the device and any related patents.  ii) Since the website represents the Sanyu Africa Research Institute (SAfRI), the affiliations between this institute and the author(s) must be clarified. | Thank you for this important comment, and apologies for overlooking this important issue.  We have clarified the affiliations with the Sanyu Africa Research Institute (SAfRI) and have updated the funding and conflict of interest sections at the end of the document. | 647-663 |
| **Reviewer #1** |  |  |
| 1. How they arrived at a sample size estimation of 150 observations, and the entry point thereof.   How much power these 15 observations afforded to the study. | Thank you for these comments regarding sample size estimation. The target sample size was 150 neonatal resuscitation observations. The sample size was based from local hospital data. Mbale Regional Referral Hospital conducts approximately 9,000 deliveries annually. Based on hospital estimates, about 6% of newborns (approximately 540) require resuscitation annually. Assuming that 50% of these would be eligible for bedside resuscitation using the BabySaver (n = 270), and considering a six-month data collection period, we aimed to observe around 135 resuscitation events. To account for potential missed observations, we added a 10% inflation, resulting in a final target sample size of 150 observations, with 75 in each phase (pre- and post-implementation). As a feasibility study, no formal sample size calculation was needed, but we considered that a sample of 75 in each group would be adequate to detect an increase in bedside resuscitation from 10% baseline to 30% after implementation (5% significance level and 90% power). | 155-162 |
| 2. From the Figure 1, flow chart the entry point is the total neonatal resuscitations during each phase, of whom 150 consented in both the pre and post arms; and only 75 of each were included in the final analysis. So, how did the authors select the specific 75 observations in each arm out of the 150 | Thank you for pointing this out. We sincerely acknowledge the concern and appreciate the opportunity to clarify this issue. The initial figure was a misrepresentation resulting from a miscommunication from the data management team. We have reviewed our data sources thoroughly to ensure that the corrected numbers accurately reflect actual enrollment. The actual number of participants who consented and were included in each phase was 75. During the pre-implementation phase, 81 mother–infant dyads were eligible, and 75 were enrolled. In the post-implementation phase, 78 mother–infant dyads were eligible, and 75 were enrolled. We have revised the manuscript and Figure 1 to accurately reflect the total number of enrolled participants (75 per phase). | 255-263 |
| 3. Abstract: Is well written and concise | Thank you for this feedback. We are glad the abstract is well written and concise. | NA |
| 4. The narrative in the background did not allude to the fact that bedside resuscitation was already in practice pre- implementation as we find later in the results. Was this contributed by previous interventions or was this part of the standard of care? | Thank you for this important observation. We acknowledge that the background section did not explicitly state that bedside resuscitation practices, though limited, were already occurring prior to the implementation. The WHO recommends that delayed cord clamping should be practiced for every newborn and this has been emphasized through webinars prior to the intervention. However, bedside resuscitation for asphyxiated newborns was not routine and was constrained by the lack of appropriate equipment at the mother’s bedside. This study focused specifically on resuscitation of asphyxiated newborns, where the standard approach was still early cord clamping and transfer to a separate resuscitation area. We have included it in the study setting. | 111-114 |
| 5. It would be worthy to cite (Ditai J et al, 2021) who developed the BabySaver in the same hospital and inform the reader if its use had been adopted or not and what were the concerns and how have these been addressed in the current study? or if this is the first feasibility study on the BabySaver? | Thank you. We have cited Ditai J et al., 2021, and have clarified that this is the first feasibility study of BabySaver and NeoBeat together under the study strengths. Previous work focused on development and design. | 85 and 572 |
| 6. Grammar: Paragraph line 8 .... This practice prevents the practice of delyaed cord clamping............ The double use of practice in the same sentence can be improved | Thank you for highlighting this, we have revised it to; “*This practice hinders delayed cord clamping and may also cause maternal anxiety and denial.”* | 81 |
| 7. Problem statement and Objectives: Were well stated | Thank you for this feedback. We are happy that the problem statement and objectives are clear and well-stated. | NA |
| 8. Paragraph 4 in the Background section   Justification: This can be enhanced by citations that show the potential benefits of beside resuscitation in terms of morbidity indicators (early initiation, improved Apgars, decreased neonatal morbidity) and not just the delayed cord clamping benefits [REF 13-15]. | We have revised and added citations demonstrating reduced neonatal morbidity (improved Apgar scores, and early initiation). | 88-89 |
| 9. Line 5..... The hospital has one labour ward theatre ....... this phrase can be changed to an obstetric theatre a terminology which will resonate with most readers. | Thank you for the suggestion, this has been done. | 106 |
| 10. Sample size estimation and sampling needs clarification as mentioned in the above summary | Thank you for your comment. We have revised the manuscript to provide a more detailed explanation of how the sample size of 150 observations (75 per phase) was estimated. The estimation was based on institutional data indicating approximately 9,000 annual deliveries at Mbale Regional Referral Hospital, with 6% requiring resuscitation (n = 540). Assuming 50% eligibility for bedside resuscitation, this gave an estimated 270 eligible cases over one year. Over the six-month study period, we expected around 135 eligible cases. We inflated this by 10% to account for missed or incomplete observations, resulting in a final target sample of 150 observations.  Eligible participants were consecutively enrolled until the required sample size of 75 per phase was achieved. As a feasibility study, no formal sample size calculation was needed, but we considered that a sample of 75 in each group would be adequate to detect an increase in bedside resuscitation from 10% baseline to 30% after implementation (5% significance level and 90% power). | 155-162 |
| 11. Variables   Paragraph 2. ........A clarification between the baseline socio-demographic characteristics versus the independent and dependent variables is warranted to be able to interpret the tables correctly. Table 3 variables are dependent on whether a pre or post implementation approach was adopted and not the reverse. | Thank you for this insightful comment. We agree that the distinction between baseline socio-demographic characteristics and the study’s independent and dependent variables needed clarification. We have revised Table 3 to show the study phase (pre or post) as the independent variable, and place of resuscitation as the dependent variable.  Additionally, we have clarified that the baseline socio-demographic characteristics presented in Table 1 serve a descriptive purpose to compare participant groups across study phases, rather than acting as predictors in the analysis.  We have included study phase as one of the explanatory variables. We have chosen to use the explanatory variables | 290  174-176 |
| 12. Data collection: Was well elaborated | Thank you, we appreciate your acknowledgment. | NA |
| 13. Data analysis For, both phases was done well, using the appropriate/relevant statistical methods for the study design | Thank you, we are pleased that the data analysis approach was found appropriate and well done. | NA |
| 14. Ethical consideration for intrapartum research was duly followed and clearance was obtained through the right pathways. | Thank you for acknowledging our efforts in adhering to ethical standards for intrapartum research. | NA |
| 15. Results Overall the results were well presented | We appreciate your observation and are glad that the results are well presented. | NA |
| 16. Study profile   Line 5. in the post intervention arm only 152 mother-infant dyads were eligible not 155 and shown in Figure 1 | Thank you for the observation. We have reviewed and corrected the study profile accordingly | 264-265 |
| 17. Table 4 Comparison of the ...... birth outcomes pre and post implementation of the bedside neonatal resuscitation did not show any difference in the condition of the neonate at 24 and 48 hours. This was not discussed by the authors. Is there any explanation for this? Does it call for long-term follow up research? | Thank you for this valuable comment. We acknowledge the omission and have now addressed this in the Discussion section. *However, we did not observe differences in the condition of the infant at 24 and 48 hours. This may be due to the limitations of short-term outcome measures, which may not adequately reflect the benefits of timely bedside resuscitation.* | 517-519 |
| 18. The qualitative findings were well presented and the use of an existing theoretical framework was ideal in streamlining the interpretations of the arising themes, although there was some overlap in the some constructs. | Thank you for your comment. | NA |
| 19. The suggested modifications added great value to the qualitative findings allowing the end user to contribute towards the quality improvement of the intervention | Thank you for this feedback. | NA |
| 20. Discussion: Was brief but concise and focused on the responding to the main objective. | Thank you for this comment. | NA |
| 21. Typo ........   Paragraph 1 Line .5...... fewer babies needed further care post resuscitation  Paragraph 2 Line 16......Several studies including systematic reviews | We have revised these statements to: “Fewer babies required additional care post-resuscitation.” and, “Several studies, including systematic reviews…” as guided. | 489 and 513 |
| 22. Limitations: The use of the same researchers for the IDI may indeed have biased the participants. Is there a reason different research assistants were not allocated for this phase? If so, how was the potential for bias mitigated? | Yes, this was a possible source of bias However, we mitigated bias by training researchers on qualitative data collection techniques including the practice of reflexivity. We have updated this under discussion section. | 584-586 |
| 23. Conclusions and recommendations: We appropriate and derived from the study findings. | Thank you for your comment. | NA |
| 24. References: These were appropriately selected but it would help to know what reference style the authors used to assess for consistency? | We used Vancouver referencing style throughout and have ensured consistency. | NA |
| **Reviewer #2** |  |  |
| 1. This is a well written and important work. I believe the conclusions are sound and based on the research which is well described. I have a few important queries/observations, and then a number of less important minor issues. | We sincerely thank the reviewer for the positive and encouraging feedback on the overall quality, relevance, and clarity of our work. | NA |
| 2. I think the issues around the sample size need to be better clarified. What is the potential change in outcome that was used to calculate the sample size? Improved resuscitation at the bedside? And if so, where does the assumption of a 50% change come from? What alpha/power was used? | Thank you for this important observation. We acknowledge that further clarification was needed regarding the assumptions underlying the sample size calculation. We have revised it and now reads; *Based on the hospital’s annual number deliveries (9,000), the percentage of neonates (6%) that require resuscitation in Mbale Regional Referral Hospital (local data) (9000*0.06=540). Given the assumption that 50% of eligible neonates would receive bedside resuscitation (540*0.5=270). Over a period of six months, we estimated to observe (270/2=135). We inflated the sample size by 10 percent to cater for missed observations. The required sample size was 150 observations, with 75 in each phase. We assumed a two-sided confidence interval of 95%, and 99.9% power using a before and after approach. We consecutively enrolled the mother-infant dyads until the required sample size was accrued in each phase.* As a feasibility study, no formal sample size calculation was needed, but we considered that a sample of 75 in each group would be adequate to detect an increase in bedside resuscitation from 10% baseline to 30% after implementation (5% significance level and 90% power). | 156-164 |
| 3. I find the graph documenting differences in temperature very interesting. I am not clear whether the initial temperature is at the time of birth or at 1 minute. It would seem odd for there to be a difference in temperature at the time of birth. But – before intervention- it seems the initial temperature (? at 1 min) was 33 degrees? That seems noteworthy and I wonder if it is accurate? | Thank you. Initial temperature was recorded within the golden minute. This observation of low temperature may reflect heat loss during transfer to the resuscitation after early cord clamping in the pre-implementation phase. However, in the discussion, we postulate that babies resuscitated with an intact cord receive a transfusion of warm blood form the mother and may have a better regulation of their temperature. | 520-527 |
| 4. It is reported that the duration of resuscitation was shorter post intervention. Were there specific objective criteria to determine when resuscitation stopped? Should these be listed? | We were able to tell the duration of resuscitation more accurately using the Liveborn app, that we used to capture time stamps in both the pre and post implementation phase. | 187-189 |
| **Reviewer #3** |  |  |
| 1. I have a few questions around table 4. The footnotes all say “adjusted for antepartum haemorrhage and malpresentation”. I suspect this is a typo. Perhaps the correct footnotes may answer some of my questions. | We apologize for this oversight. This has been clarified under the statistical analysis section, antepartum haemorrhage and malpresentation were adjusted for, in all the models. | 224-225 |
| 2. Under outcomes of “the resuscitation process” it mentions early neonatal death. This seems less than the number of deaths at 24 h- does this line refer to babies who died in the resuscitation process? That should be made clear. And there were infact an increase in the number of deaths- albeit a small one from 0 to 3. But no p value given- I would think this is important, from my calculatiosn using a Fischers exact I get 0.244. I think this is worth mentioning in the text, even if it is not significant. I am also wondering why the total number of babies in the 24 h and 48h intervals does not add up to 75. | Thank you for the comment. The early neonatal deaths mentioned under the resuscitation process refer to babies who died during or immediately following resuscitation before admission to the postnatal ward.  Under condition at 24 hours, for deaths, no p-value is reported because the numbers were one-sided and the statistical model did not yield results for this outcome; we did not perform Fisher’s exact test. We have clarified this point in the manuscript text.  Regarding the total number of infants assessed at 24 and 48 hours: fresh stillbirths and those who died before reaching the 24-hour timepoint were excluded from these analyses. As a result, the denominators for the 24-hour and 48-hour outcomes are slightly lower than the full sample of 75. We have clarified this exclusion criterion in both the results and footnotes of Table 4. | 325 |
| 3. In the methods section there seems to a semi- repetition of a sentence around the use of a phenomenological method. | Thank you for highlighting this, we have revised it accordingly. | 100 |
| 4. There is mention of a resuscitation checklist in the methods- and then in the data it is mentioned that the liveborn LGH tool is used- if these are the same, I may mention the LGH tool in the methods. | Thank you for this comment, we used both the checklist and the Liveborn LGH app to collect data. Liveborn LGH app was used to collect data regarding the resuscitation process including the time of starting and stopping the resuscitation. | 187-190 |
| 5. In the table about characteristics of the mothers, some are classified as housewives and others as unemployed. What was the distinction used to separate these two categories? | Thank you for this observation. We think that we can combine the two without losing any meaningful information. This has been done, see Table 1 | 272 |
| 6. I was often intrigued by what the products may look like- is there a possibility to show a photo of them, ideally in use? Or set up for use? | We have added a figure with photos of the BabySaver and NeoBeat setup in the methods section. | 155 |
| 7. In the qualitative section, it is mentioned that 63% of the mothers had secondary education, but for the group as a whole I see it was more like 40%- should this be mentioned? | Thank you for pointing out this discrepancy. We have reviewed and corrected the figure to 46.7% for the qualitative subgroup, the mothers. | 342 |
| 8. I also feel that the slightly different training for staff in the pre and post intervention period may have had some impact on the outcomes (ie not just the gadgets themselves) and I think that might need to be mentioned. | Thank you for this valuable insight. We agree that differences in training provided to staff before and after implementation may have influenced the study outcomes. We have highlighted it in the discussion as a possible contributing factor to the observed changes. | 525-526 |
