# Supplementary material for: Feasibility and acceptability of using the BabySaver resuscitation platform and NeoBeat together for neonatal resuscitation in a low-resource setting: A pre-post implementation study
Source: PLoS One. 2025 Dec 4;20(12):e0337088. doi: 10.1371/journal.pone.0337088 (PMC12677507; doi:10.1371/journal.pone.0337088)
Supplement: Attachment — Submitted filename: DCC_response to reviewers_2.docx [file pone.0337088.s004.docx]

**Re: “PONE-D-25-16948 Feasibility and acceptability of using the BabySaver resuscitation platform and NeoBeat together for neonatal resuscitation in a low-resource setting: A pre-post implementation study”**

Thank you for revising our manuscript.

Below is our point-by-point response to each comment.

Thank you.

| **Comment** | **Response to comment** | **Line number** |
| --- | --- | --- |
| **Reviewer #1** |  |  |
| 1. Thank you, the author(s) have addressed the comments stipulated and they have cleared out the sample size and sampling queries which were previously confusing. Similarly, the revision of Table 4 was a crucial step in clarifying the key outcomes,   1, one minor comment in the background line 81 .................. This practice hinders delayed cord-clamping and may also cause mother anxiety and denial CHANGE TO..........to maternal anxiety | Thank you for this feedback.  Thank you for the suggestion, we have revised this sentence and it now reads; “This practice hinders delayed cord-clamping and may also cause maternal anxiety and denial.” | 81 |
|  |  |  |
| 2. In the results, Line 311-312, the narrative on the lack of a difference in outcome at 24 hours and 48 hours should still be documented. This has been rightfully included in the discussion, though it is my opinion that HIE is an evolving condition and the outcome at 24 and 48 hours may be influenced by affection of other organ systems and the management thereof; and is not limited to the initial resuscitation results. | Thank you for pointing this out. We have added a statement under results highlighting that: “There was no difference in the condition of the infant at 24 and 48 hours between the pre- and post- implementation periods.” | 314-315 |
| **Reviewer #2** |  |  |
| 1. Thank you for addressing my previous comments.  I still feel the claim that baby's axillary temperature at 1 min was 33.5 degrees warrants a comment in the discussion. I strongly suspect this was a measuring error. Even babies who are actively cooled with icepacks take 30 min to drop their temperature that far. I find it hard to accept that a baby no more than 1 min old, who presumably had a normal temperature 1 min ago, could drop their temperature to 33 degrees in 1 minute. | Thank you for this comment.  We suspect hat this was due to the fluid on the babies’ foreheads immediately after birth. We have included this as a limitation and state it as below:  “One limitation of infra-red temperature assessment is the tendency to underestimate readings in the presence of moisture. We took our first temperature measurement immediately after birth, before drying the baby. This could have resulted in a systematic underestimation of the temperature reading at 1 minute. However, this error would have been similar in both the pre-implementation and post-implementation period and we suspect that this did not affect the difference between the two periods.” | 260-265 |
