# Supplementary material for: Feasibility and acceptability of using the BabySaver resuscitation platform and NeoBeat together for neonatal resuscitation in a low-resource setting: A pre-post implementation study
Source: PLoS One. 2025 Dec 4;20(12):e0337088. doi: 10.1371/journal.pone.0337088 (PMC12677507; doi:10.1371/journal.pone.0337088)
Supplement: Attachment — Submitted filename: DCC_response to reviewers_3.docx [file pone.0337088.s005.docx]

**Re: “PONE-D-25-16948 Feasibility and acceptability of using the BabySaver resuscitation platform and NeoBeat together for neonatal resuscitation in a low-resource setting: A pre-post implementation study”**

Thank you for revising our manuscript.

Below is our point-by-point response to each comment.

Thank you.

| **Comment** | **Response to comment** | **Line number** |
| --- | --- | --- |
| **Editor** |  |  |
| Correct the conflict of interest statement in the standardised table before the manuscript so that it aligns with the conflict of interest statement at the end of the manuscript (the receipt of royalties is a potential conflict of interest). | Thank you for your comment, this has been done. |  |
| **Reviewer #2** |  |  |
| Thank you for working on the previous comments which have added great clarity to your work thus far.  I have very minor comments which you can consider for your final version  Few corrections  Abstract  Line 45: the median time to successful resuscitation was shorter 8 versus 5 minutes, [I think should be the reverse '5 versus 8 minutes]'   1.   Background  Line 66: hindered by a lack of training and suitable and functional equipment [ 'and suitable or functional resuscitation equipment '] may be used to avoid the double and in the sentence  Study population  Line 116 admitted to the neonatal unit ['for'] assessment and ongoing management... the word for is missing  Study procedures  Lines 122 and 136 ['obstetric theatre'] rather than labour ward theatre  One inquiry  Background  Line 85:Just wanted clarity why it is said the BabySaver has not been clinically tested whilst it had undergone phase 1 and 11 clinical testing and part of this was done in Uganda? | Thank you for this feedback  Thank you, these grammatical errors have been revised accordingly.  45  68  119  125, 136  Thank you for the comment. There had been no clinical testing conducted prior to this study. However, the design and development of the BabySaver was partly done in Uganda. | 260-265 |
