# Supplementary material for: Feasibility and acceptability of using the BabySaver resuscitation platform and NeoBeat together for neonatal resuscitation in a low-resource setting: A pre-post implementation study
Source: PLoS One. 2025 Dec 4;20(12):e0337088. doi: 10.1371/journal.pone.0337088 (PMC12677507; doi:10.1371/journal.pone.0337088)
Supplement: Attachment — Submitted filename: DCC_response to reviewers_4.docx [file pone.0337088.s006.docx]

**Re: “PONE-D-25-16948 Feasibility and acceptability of using the BabySaver resuscitation platform and NeoBeat together for neonatal resuscitation in a low-resource setting: A pre-post implementation study”**

Thank you for revising our manuscript.

Below is our point-by-point response to each comment.

Thank you.

| **Comment** | **Response to comment** | **Line number** |
| --- | --- | --- |
| **Editor** |  |  |
| Please correct the competing interests’ section on page 5 of PONE-D-25-16948R3 still reads, "The authors have declared that no competing interests exist." Please correct this to align with the statement in the manuscript which reads,  "ADW was one of the co-inventors of the BabySaver tray. The intellectual property is held by his employer, the University of Liverpool, but the rights for Africa were sold to the Sanyu Africa Research Institute for £1 in 2019 so that they could take forward its development and  distribution in Africa. ADW holds no personal rights to the IP, but there is a royalty sharing scheme at UoL whereby any future royalties would be shared with him." | Thank you for your comment. We are unable to locate where to update the Competing Interests section in the submission portal. We previously contacted PLOS Customer Care and were advised to include the updated statement in the cover letter, after which the PLOS team would make the update on our behalf.  Their response was as follows:  “Thank you for your query. The Competing Interests statement can't be edited on your end past initial submission, so please include your new statement in your cover letter and we will update this for you.”  We added the updated statement to the cover letter.  We kindly seek your guidance on how best to proceed in this regard. |  |
